# Neural Representations of the Self and the Mother for Chinese Individuals

**DOI:** 10.1371/journal.pone.0091556

**Published:** 2014-03-10

**Authors:** Gaowa Wuyun, Min Shu, Zhijun Cao, Wei Huang, Xin Zou, Sheng Li, Xin Zhang, Huan Luo, Yanhong Wu

**Affiliations:** 1 Department of Psychology, Peking University, Beijing, China; 2 Chinese Academy of Sciences, Beijing, China; 3 Learning and Cognition Lab, Capital Normal University, Beijing, China; University of Bologna, Italy

## Abstract

An important question in social neuroscience is the similarities and differences in the neural representations between the self and close others. Most studies examining this topic have identified the medial prefrontal cortex (MPFC) region as the primary area involved in this process. However, several studies have reported conflicting data, making further investigation of this topic very important. In this functional magnetic resonance imaging (fMRI) study, we investigated the brain activity in the anterior cingulate cortex (ACC) when Chinese participants passively listened to their self-name (SN), their mother’s name (MN), and unknown names (UN). The results showed that compared with UN recognition, SN perception was associated with a robust activation in a widely distributed bilateral network, including the cortical midline structure (the MPFC and ACC), the inferior frontal gyrus, and the middle temporal gyrus. The SN invoked the bilateral superior temporal gyrus in contrast to the MN; the MN recognition provoked a stronger activation in the central and posterior brain regions in contrast to the SN recognition. The SN and MN caused an activation of overlapping areas, namely, the ACC, MPFC, and superior frontal gyrus. These results suggest that Chinese individuals utilize certain common brain region in processing both the SN and the MN. The present findings provide evidence for the neural basis of the self and close others for Chinese individuals.

## Introduction

The concept of the self is constructed as the subject of one’s own being or as an object distinct from others [1]. The neural basis of the self has been extensively investigated. A series of studies have revealed that cortical midline structures (CMS), such as the anterior cingulate cortex (ACC), medial prefrontal cortex (MPFC), and posterior cingulate cortex (PCC), reflect self-referential processing and self-reflection [1], . In most studies, the subjects were presented with two types of stimuli with contrasting self-relevance, namely, self-relevant and self-irrelevant (unknown other) information. However, these studies may have been confounded by the effect of familiarity because self-related material is intrinsically familiar. Self-information can be distinguished effortlessly from unknown other information. Moreover, recent studies have developed control conditions by introducing close others and examining whether the neural representations of close others are similar to or different from the concept of the self [5], [6], [7], [8]. Because close relationships also play an important role in an individual’s life, the brain may generate unique neural representations of close others [9]. Thus, the relationship between the self and close others in neural representations is considered an interesting topic, particularly in social neuroscience.

Many studies have investigated this topic and shown consistent results [5], [6], [7], [10], [11]. Employing the self-reference (SR) paradigm, Seger et al. (2004) reported that the self and close other judgments have similar neural activation patterns in the MPFC. Ochsner et al. (2005) failed to observe differences in MPFC activation between the self and close other judgments. Similarly, Zhu et al. (2007) found that the mother judgment task evokes MPFC activation in Chinese participants but not in Western participants. Thus, previous studies have interpreted the MPFC activation as an indication for a shared neural representation of the self and close others. However, whether the overlap in activation between the self and close others reflects a common functional mechanism or is a result of poor sensitivity in resolving differences in the MPFC remains an open question.

Recent studies have indicated that the observed involvement of the MPFC region cannot be fully accounted for by self-specificity [4], [12]. The MPFC only plays a role in the processing of personally relevant stimuli [13]. Overlapping MPFC activation indicates that judgments about the self and others may share a common cognitive process, such as theory of the mind or mentalizing [14]. In contrast, the anterior cortical regions (the ACC and anterior insula) are more commonly associated with self-specific stimuli [15], [16], [17]. A recent study by Qin et al. (2012) showed an overlapping activation in the MPFC when the passive processing of the self-name (SN) was compared with that of close others’ names. However, different activations by the SN and familiar names were detected in the posterior ACC. The clear divide between self-specificity and familiarity in the ACC and MPFC strongly suggests that the posterior ACC is more sensitive to self-specific stimuli [4]. However, most studies used a self-referential task that required participants to explicitly perform a trait-judgment. Thus, participants were required to complete several forms of cognitive processing, such as judgment, evaluation, and categorization, raising the possibility that the neural activity would be changed by these potential confounding variables.

In the current study, we chose a name-processing paradigm to explore the neural basis of the self and close others. Through an acoustic channel, this paradigm presents the SN, close others’ names, or unknown names; the names are presented randomly and repeatedly while the brain is scanned to determine the areas that are activated [2], [3], [5]. Tacikowski et al. (2012) conducted a study employing a cross-modal pattern (visual and auditory) and diverse close others’ names to investigate the neural basis of the self and significant persons. They required participants to consciously perform a familiarity judgment, raising the possibility that the participant may intentionally strengthen the connection between the SN and the close others’ names to improve their task performance. Such an explicit task cannot capture the specific name processing that occurs in everyday life because being asked about the familiarity of one’s name is relatively unusual. Qin et al. (2012) adopted a modified version of the name-processing paradigm with an orthogonal (i.e., non-self-related) task, in which participants were asked to passively listen to names without explicit self-related judgment [4]. To construct such a task, the participants in the present study were asked to make self-irrelevant judgments about a specific probing stimulus.

The close others that Tacikowski et al. (2012) and Qin et al. (2012) adopted in their studies involved both parents’ names and friends’ names. However, different types of significant others may relate to the self in different ways and may therefore correspond to different neural correlates. For example, Wang et al. (2011) reported that the neural representations of the mother, father, and friends are different and that the mother has a unique effect on Chinese individuals [5], [8]. This type of mother effect on Chinese individuals is supported by numerous studies that show comparable brain activity for trait judgments of the self and the mother [10]. In Chinese culture, taking care of infants and children is mainly the responsibility of the mother. Therefore, children may show a stronger emotional relationship with their mothers. In addition, based Markus and Kitayama’s classification [18], [19], they proposed that Eastern Asian culture accentuates the interdependent construal of the self; they also indicated that the self has some interactions with others and that significant others may be embraced into the self structure. Thus, the relationship between the self and the mother would be closer and more special for Chinese individuals.

The current study aimed to investigate whether the neural representation of the mother occurs in a region overlapping that of the self. To achieve this goal, we compared brain activations while processing the SN and MN during passive name recognition. The self-expansion model indicates that individuals may absorb the perspectives and characteristics of close others into the self to maintain their relationships [20]. If Chinese individuals make an especially strong connection with their mothers, we can expect an important overlap in the activations in not only the MPFC but also the ACC across these two conditions. Conversely, if the self is always represented by a distinct mechanism relative to the mother, the enhanced activity should be found in the ACC during the SN encoding.

## Methods

### Ethics statement

The research reported in this manuscript has been approved by the Human Subjects Review Committee of Peking University, written informed consent was given prior to participation in this study.

### Participants

Twenty-six healthy, right-handed college students from Peking University and Tsinghua University participated in this study. Sixteen participants were male. All participants had normal hearing and had not changed their names in at least 10 years.

### Stimuli and procedure

Three types of names were employed, namely, the SN, the MN, and the unknown names (UNs). These names consisted of three words. The full name (i.e., the first and second names) was adopted in the current study because it is formal and may attract more attention than the first name alone. Moreover, having the same full name as others is less likely than having the same first name alone; thus, the full name could be self-related. In the experiment, the gender of each UN was matched with that of the SN, and the participants of the same gender listened to the same UNs. Each participant was presented with five UNs, with one UN serving as the probing stimulus. The participants were required to press the button when they heard either of the two probing stimuli (e.g., “Bao Jianwei” or “Qiu Xuemei”). These UNs were strictly selected, with controlled familiarity and recognizable gender information. The voice stimuli of two speakers (one male and one female) were recorded. The genders of the speakers and the participants were matched. Thus, the participants of the same gender listened to the same speaker’s voice. Cool Edit Pro was used to edit the voice stimuli, keeping them within the same duration (900 ms) and same voice power (average root-mean-square power = −23 dB).

**Figure 1:**
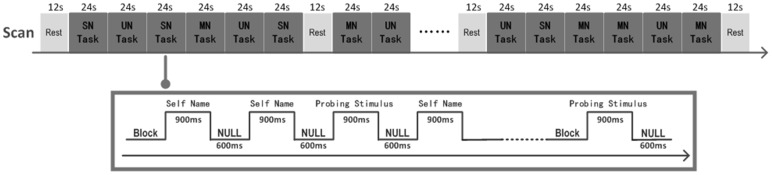
Schema of the design of one scan of the current study.

A block design was adopted using name (i.e., SN, MN, and UN) as the independent variable and recorded brain activation as the dependent variable. Two functional scanning sessions were included. Each functional scan lasted for 10.6 min, and the total time was approximately 20 min. Each scan contained 24 blocks, with 8 blocks for each name condition. These blocks were presented randomly. Each scan involved five rest stages that lasted for 12 s; one rest stage was presented at the beginning of the scan, and the other four were presented at the end of every six blocks (Figure 1). Each block lasted for 24 s for 16 trials. Among these trials, fourteen presented the experimental stimuli (i.e., SN, MN, and UNs), and two presented the specific probing stimuli (e.g., “Bao Jianwei” and “Qiu Xuemei”). These trials were presented randomly. Each trial lasted for 1,500 ms and involved a 900-ms voice stimulus followed by a 600-ms blank. Each participant listened to the SN, MN, and UNs a total of 224 times.

During scanning, the participants listened to the stimuli using a headset connected to a computer. They were required to press a button when they heard either of the two probing stimuli. This task aimed to control the attentional effects by forcing participants to respond to low-rate probing stimuli, thereby facilitating the examination of automatic processing for name recognition [21], [22].

The present study demonstrated a “practice effect”, as the mean reaction time (RT) to the probing stimuli was shorter in Scan 2 than Scan 1 [*F*(1, 22) = 10.41, *P*<0.01]. The whole-brain analysis showed the dissociation between the two scans, with the SN and MN provoking stronger activation in the frontal areas in Scan 1 and a faded signal in these areas in the two conditions in Scan 2. Golby et al. (2001) also found that novel stimuli, rather than repeated ones, induce stronger activation in the bilateral prefrontal areas. In the current study, only one name was included in the SN and MN conditions, and four different names were presented in the UN condition. As the experiment proceeded, the repetition of the SN and the MN became considerably greater than that of the UN, turning the UN into a more novel stimulus than the SN and MN. Therefore, as a consequence of this practice effect, we only present the analysis of the first scan.

### fMRI data acquisition and analysis

Scanning was performed at the Beijing MRI Center for Brain Research on a 3-T Siemens Trio Scanner with a standard head coil. Twelve channels were used during the scanning in the head coil. Thirty-two transverse slices of functional images covering the entire brain were acquired using a gradient-echo echo-planar image (repetition time [TR] = 2000 ms, echo time [TE] = 30 ms, flip angle = 90°, slice thickness = 3 mm, field of view [FOV] = 240 mm, matrix size = 64 mm × 64 mm, spatial resolution = 3.75 mm×3.75 mm×3.75 mm). Anatomical images were attained using a 3D T1-weighted fast spoiled gradient sequence (TR = 2530 ms, TE = 3.37 ms, slice thickness = 1.3 mm, FOV = 256 mm, matrix size = 256 mm×256 mm, plane resolution = 1.0 mm×1.0 mm).

The imaging data were analyzed using Analysis of Functional NeuroImages (AFNI) [23]. The functional images were realigned to the first scan to correct for head movement between scans and were co-registered with each participant’s anatomical scan. The functional images were then normalized into a 3.5 mm×3.5 mm×3.5 mm Talairach space [24]. The normalized data were spatially smoothed by a Gaussian filter with the full-width at half maximum parameter set to 4 mm.

The imaging data utilized a block function for model building. The parameter estimate for each task was obtained by calculating the general linear model based on the hemodynamic response function. The parameter was extracted from each subject’s data for the group analysis. A two-factor ANOVA, with trial type (i.e., SN, MN, and UN) and subjects as the within-subject and random factors, respectively, was adopted.

Signal data from the implicit name recognition paradigm task were weak. Therefore, for the whole-brain analyses, we performed a multiple-test correction using the 3dClustSim program in AFNI, which follows a family-wise approach based on a Monte Carlo simulation for calculating the minimum necessary cluster size to assure a family-wise error rate of *p*<0.05 (http://afni.nimh.nih.-gov/pub/dist/doc/program_help/3dClustSim.html). Only regions with a cluster size of more than 108 contiguous voxels were considered significantly activated regions.

To investigate the relationship between the neural correlates of the SN, MN, and UN processing, this study conducted a conjunction analysis based on the whole-brain analysis. We calculate the logical AND of two main comparisons (SN vs UN and MN vs UN) [25]. Only the clusters large enough to survive the FWE correction will be used in the conjunction analysis.

## Results

A repeated-measures ANOVA of the mean RTs and the corrected accuracy rate (hits minus false alarms) were conducted with name type (i.e., SN, MN, and UN) as the independent within-subjects variable. No significant main effect of the name type was found, regardless of whether RT or corrected accuracy rate was employed as the dependent variable.

The SN and MN conditions were first contrasted with the UN condition to identify the brain areas respectively involved in the processing of the self and the mother. The results show that compared with the UNs, the SN induced a significant activation in the bilateral MPFC (t_25_ = 4.68, *p*<0.001), ACC (t_25_ = 3.93, *p*<0.001), left inferior frontal gyrus (IFG) (t_25_ = 4.25, *p*<0.001), left middle temporal gyrus (t_25_ = 3.93, *p*<0.001), and left temporoparietal junction (TPJ) (t_25_ = 3.85, *p*<0.001) (Table 1). Compared with the UNs, the MN induced a significant activation in the bilateral MPFC (t_25_ = 4.74, *p*<0.001), ACC (t_25_ = 2.42, *p*<0.05), left paracentral lobe (t_25_ = 4.45, *p*<0.001), left PCC (t_25_ = 2.65, *p*<0.05), and left TPJ (t_25_ = 4.36, *p*<0.001) (Table 1). Moreover, the SN only invoked the bilateral superior temporal gyrus in contrast to the MN (left: t_25_ = 4.46, *p*<0.001; right: t_25_ = 5.26, *p*<0.001); the MN invoked the occipital lobe (t_25_ = 5.24, *p*<0.001), bilateral fusiform (left: t_25_ = 3.91, *p*<0.001; right: t_25_ = 4.72, *p*<0.001), left prefrontal lobe (t_25_ = 4.80, *p*<0.001), right parahippocampal gyrus (t_25_ = 4.33, *p*<0.001), PCC (t_25_ = 4.57, *p*<0.001), bilateral supplemental motor area (left: t_25_ = 4.69, *p*<0.001; right: t_25_ = 6.28, *p*<0.001), and left parietal cortex (t_25_ = 5.07, *p*<0.001).

**Table 1:** Brain activations shown in various contrasts (p< 0.05, two-tail).

	Volume	BA	X	Y	Z	t value	Region
SN>UN							
	300	8,9,24,32	−5.2	48	27.5	4.68*	MPFC and ACC
	212	45	−43.8	16.5	17	4.25*	Left IFG
	115	22	−54.2	−39.5	3	3.93*	Left MTG and TPJ
MN>UN							
	827	6,8,9,24,32	−22.8	44.5	34.5	4.74*	MPFC, ACC
	201	5,6,7	8.8	−32.5	52	4.45*	Left paracentral lobe
	94	7	−15.8	−43	27.5	4.517	Left PCC
	63	40	−47.2	−46.5	34.5	4.36	Left TPJ

Note: *X*, *Y*, and *Z* are Talairach coordinates; MPFC = medial prefrontal cortex; ACC = anterior cingulate cortex; IFG = inferior frontal gyrus; MTG = middle temporal gyrus; TPJ =  temporoparietal junction. * corrected for multiple comparisons.

**Figure 2:**
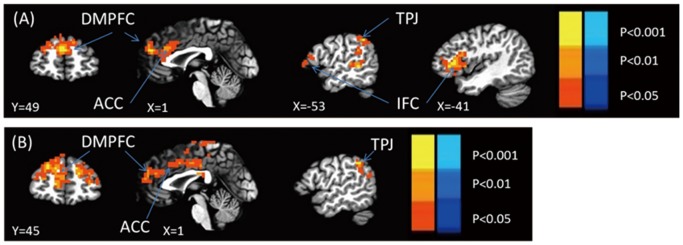
Contrasting brain activation patterns elicited by different name types. (A) SN>UN; (B) MN>UN.

**Figure 3:**
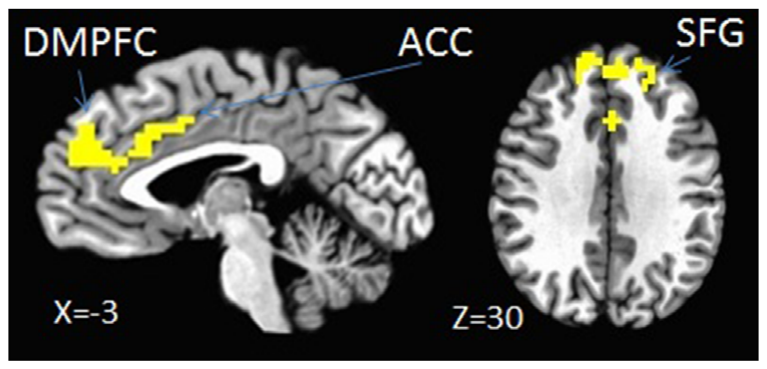
Conjunction analysis of the brain activation patterns of SN and MN compared with those of UN reveals that the MPFC, ACC, and SFG are activated both in SN and MN conditions.

According to the whole-brain analysis, the SN and MN had similar neural representations, especially in the CMS (i.e., the MPFC and ACC) (Figure 2). To further investigate the similar neural representations of the SN and MN, a conjunction analysis was conducted. The results indicate a shared neural representation in the bilateral MPFC, ACC, and bilateral SFG (Table 2, Figure 3).

**Table 2:** Brain activations shown in the conjunction analysis (cluster level).

Volume	X	Y	Z	Region
93	−5.7	43.6	30.9	MPFC
38	0.1	19	30.3	ACC
34	−23	47.8	27.9	left SFG
16	12.7	49.1	31	right SFG

Note: *X*, *Y*, and *Z* are Talairach coordinates; MPFC = medial prefrontal cortex; ACC = anterior cingulate cortex; SFG = superior frontal gyrus.

## Discussion

In this study, we employed a name-processing paradigm and compared the neural correlates involved in processing the SN and MN. In brief, the results show that compared with UN processing, SN processing was associated with a robust activation in a distributed bilateral cortical network, including the CMS (i.e., the MPFC and ACC), IFG, and temporal structures (i.e., the superior and middle temporal gyri). MN processing was associated with the bilateral MPFC, ACC, left paracentral lobe, left PCC, and left TPJ. Overlapping activations between the SN and MN conditions were found in the ACC, MPFC, and bilateral SFG. Furthermore, the SN invoked the bilateral superior temporal gyrus in contrast to the MN, whereas MN recognition provoked a stronger activation in the central and posterior brain regions (i.e., the occipital lobe, fusiform, and posterior cingulate gyrus) in contrast to SN recognition. Thus, these findings helped improve our understanding of the neural correlates involved in processing the self and close others.

### Differential neural representations of the self and mother

First, compared with MN recognition, SN recognition induced a larger activation in the bilateral superior temporal gyrus. The observed activation of the bilateral superior temporal structures could be due to the engagement of the fronto-temporal network in the participants. This network is known to exert a key influence on the detection of attentional priority [26], [27], [28], implying that the increased activation in the superior temporal lobe found in the present study reflects the detection and processing of the SN. Because the SN is an intrinsic and unique self-related stimulus for every individual [29], it possesses a high priority both visually and acoustically. That is, the SN captures the attention automatically, unconsciously, and specifically as well as in a manner that is out of the individual’s control [30]. Second, in contrast to the SN, the MN showed an increased activation in the central and posterior regions (i.e., the occipital lobe, fusiform, right parahippocampal, PCC, left prefrontal, SMA, and left parietal). Previous research found that the occipital lobe, fusiform, and right parahippocampal are generally involved in the primary visual processing of the face [31], [32]. These activities are linked more to the MN than to the SN; the participants may recall more facial information about their mother during the implicit name recognition. Activation of the PCC supports the retrieval of episodic memories [4]; the left prefrontal cortex is also involved in episodic memory and sematic encoding [33], [34]. Together, the processing of the MN requires the integration of facial information, episodic memories or other types of information that constitute the individual’s sense of their mother. In contrast, the SN recognition may relate sensation within the internal context of the self or the subject’s self rather than relating external information to a separable self. Alternatively, we also found that the MN induced greater activation in the left parietal area and SMA, which have been assumed to be crucial for motor attention [35], [36] and motor preparation [37]. In the present study, the participants were required to press a button to detect the two probing name stimuli. Thus, they would create a motor plan for any non-self-stimuli, including the MN.

### Common neural representations of the self and the mother

Our observations that the SN and the MN cause similar MPFC activations are in accordance with previous studies [4], [12], [13], which found similar patterns of activation in the conditions of the SN and familiar others’ names. Previous studies suggested that the MPFC is involved in not only self-processing but also any self-related information that involves both the self and close others, such as one’s mother and best friends [4], [5], [12], [13]. The common areas of relative activation in SN and MN conditions also included the medial aspect of the lateral superior frontal gyrus (SFG). These are the area associated with meaningful processing of individual words in a number of previous studies [38], [39]. The SFG was engaged during individuals words processing, thereby suggesting that the SFG may be associated with the self and familiar others.

Most importantly, to the best of our knowledge, our current fMRI results are the first to show an overlap in ACC activation for the SN and MN during passive name processing. Consistent with previous studies [4], [40], [41], the ACC was found to be closely involved in self-specific stimuli. However, the activity in the ACC associated with the MN is inconsistent with the data from a study by Qin et al. (2012), which showed that the ACC is only activated by self-specific stimuli. Hence, our results reveal common neural representations of the mother and the self. Several possible explanations of the results may be offered. First, according to the self-expansion model, individuals may absorb the perspectives and the characteristics of close others in the self to maintain their relationships [20]. Second, some social psychological researchers have proposed that culture may engender habitual ways of processing information related to the self and important others, such as one’s mother [18], [42]. The increased ACC activation by the MN in our study further suggests that these habitual ways of cognitive processing have their own corresponding neural basis. Chinese culture is well-known to endorse collectivism and interpersonal connectedness [18], [19], which may be the reason that Chinese individuals develop shared neural representations of the self and intimate individuals in the cognitive processes. Furthermore, our findings also reflect the effects of Chinese culture and support previous findings that the mother has a particularly important meaning for Chinese individuals [5], [8]. In addition to the self-expansion model and the specificity of the Chinese culture, we also should pay attention to other potential effects, such as the familiarity factor and emotional valence. The intrinsically more familiar nature of the MN was apparent. We could effortlessly discriminate the SN and the MN from the UNs. Individuals have been found to process information about themselves and their close others with a more positive evaluation. As a consequence, the SN and MN have more positive emotional valence compared with the UNs. Although the involvement of the ACC in the MN processing was identified in the present study, it was not even considered in previous studies in which different types of close others’ names were used [4]. The differences between our findings and those of previous studies may have occurred because different types of close others may relate to the self differently; thus, different types of close others may correspond to different neural correlates [8]. Further research is needed to differentiate the effects of the mother and those of general close others.

Our study has limitations that should be considered in interpreting the results. One limitation of the present study is that we employed blocked experimental designs that produce practice effects and have low ecological validity. Designs with a long interstimulus interval and a random distribution of the stimulus are generally more efficient than block designs. It should also be noted that the main finding was based on the absence of significant differences in activation between the SN and MN. Although similar negative results have been presented for previous culture studies [5], [11], [43], [44], [45], focusing only on Chinese participants may not provide enough evidence for the role of culture. The culture of collectivism emphasizes an interdependent self-construal that includes significant others in the self-concept [18], [19]. In contrast, westerners from an individualistic culture have an independent self-construal, which excludes any others. Consequently, how culture influences the representation of close others is still unclear. To address this issue, future studies should examine differences in the neural representations of different types of close others in Chinese and Western participants.

## Conclusion

The present study was designed to investigate the neural representation of the SN and MN during implicit name recognition processing. Consistent with previous studies, we observed that the SN elicited an increased activation in the CMS and in a widely distributed bilateral network. Interestingly, we found increased activations in the central and posterior regions during the processing of the MN and an overlap in the representations of the SN and the MN in the MPFC and ACC, which extends previous findings on the neural representations of the self and close others.
